# An Unusual Initial Presentation of Diffuse Large B-Cell Lymphoma as Recurrent Syncope

**DOI:** 10.1155/2019/1082543

**Published:** 2019-12-26

**Authors:** Kunhwa Kim, Harpreet Kaur, Matthew Chan, Manju Balasubramanian, Sorab Gupta, Vinicius M. Jorge

**Affiliations:** ^1^Department of Internal Medicine, Einstein Medical Center Philadelphia, Philadelphia, PA, USA; ^2^Department of Pathology, Einstein Medical Center Philadelphia, Philadelphia, PA, USA; ^3^Department of Hematology-Oncology, Einstein Medical Center Philadelphia, Philadelphia, PA, USA

## Abstract

We describe a rare presentation of diffuse large B-cell Lymphoma (DLBCL) with recurrent episodes of syncope. During the workup for syncope, the patient was incidentally found to have an extensive mass in the left thorax, which was later diagnosed as stage 2 bulky disease DLBCL. This is the rare case of lymphoma presenting as recurrent syncope without cardiac involvement. The patient did not have any further episodes of syncope after her successful treatment of DLBCL.

## 1. Introduction

Diffuse large B-cell lymphoma (DLBCL) is the most common subtype of non-Hodgkin's lymphoma (NHL) that accounts for about 30% of adult lymphoma [1]. Patients with DLBCL can have a myriad of presentations from constitutional symptoms such as B symptoms to localized effects from a rapidly growing mass.

Malignancy presenting as syncope is rare, but a few numbers of cases have been reported in tumors involving the neck [2, 3]. So far, most of these reported cases were in patients with recurrent head and neck cancer. Syncope from DLBCL without cardiac involvement, despite its frequent cervical involvement, has been only reported in 3 cases thus far to our knowledge [4–6].

Here, we present an unusual case of a patient who presented with recurrent episodes of syncope without other complaints. During workup for syncope, the patient was incidentally found to have an extensive mass in the left thorax, which was later diagnosed as DLBCL. Other diagnostics for syncope were unremarkable. The patient did not have any further episodes of syncope after her successful treatment of DLBCL.

## 2. Case Presentation

A 52-year-old African-American female with a past medical history of type 2 diabetes mellitus, hypertension, and hyperlipidemia presented to the emergency department (ED) after a syncopal episode in her home. The patient was in her usual health state before the episode. She felt dizzy while standing up and collapsed within a few seconds resulting in unconsciousness. She regained consciousness shortly afterward with residual dizziness, but otherwise did not report any symptoms before or after this episode. No seizure-like activity or incontinence was reported. Her medications included insulin, amlodipine, lisinopril, and escitalopram. The blood glucose level was normal prior to arrival. She has been adherent to all medications as prescribed. She denied weight loss, night sweats, or fatigue. She denied trauma, alcohol abuse, or drug intake. Interestingly, a week before this event, the patient had presented to the ED after a similar near syncopal episode. During her first encounter, she was discharged home with a recommendation to be followed-up by her primary care physician (PCP) with initial workup to be negative at the time. She unfortunately did not follow with her PCP.

On presentation, her vital signs were normal: heart rate was 98 beats/minute, blood pressure was 126/60 mmHg without orthostatic changes, respiratory rate was 16, and the oxygen saturation on room air was 98%. On the initial physical exam, she was well in appearance and not in any distress. The cardiac, pulmonary, and neurologic exams were unremarkable. The laboratory evaluation was within normal range: white blood count of 9,370/*µ*L, hemoglobin of 12.8 g/dL, and platelet count of 245,000/*µ*L. Routine blood chemistries panel were normal: BUN/Cr 19/0.8 mg/dL, sodium 141, potassium 4.6, glucose 109 mg/dL, AST/ALT 12/13 units/L, LDH 173 mg/dL, and uric acid 5.0 mg/dL.

**Figure 1:**
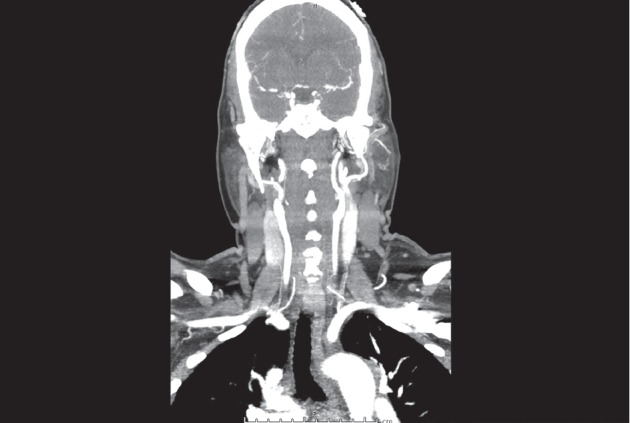
CT angiography shows patient blood circulation without tumor infiltration or encasement of the carotid body or the carotid sinus.

Her EKG showed normal sinus rhythm without ST changes. Her 2 d echocardiogram was normal with preserved ejection fraction at 55% without increased pulmonary arterial pressure. Her chest X-ray with a lateral view had a questionable enhancement in the mediastinum. The following CT head and angiography of head and neck obtained as a part of the recurrent syncope workup revealed widely patent cervical and intracranial circulation without abnormality (Figure 1). Incidentally, a soft tissue mass was found in the superior and the posterior mediastinum.

**Figure 2:**
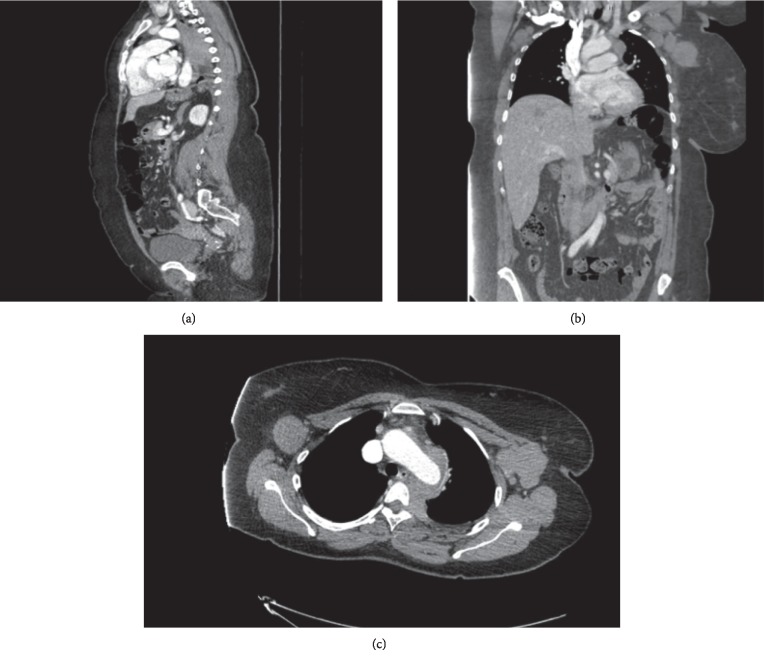
Sagittal (a), coronal (b), and axial (c) images of CT chest with contrast shows posterior mediastinal mass that encases descending aorta and extensively infiltrates to epidural space.

The patient was admitted to the general medical floor for further workup for the mediastinal mass and syncope. She underwent CT chest with contrast and MRI with and without contrast which showed an anterior and left posterior mediastinum mass measuring 4.4 × 5.7 × 13.5 cm encasing the descending aorta and multiple left-sided intercostal vessels with epidural extension into the left-sided neural foramina from T4-5 through T6-7 (Figure 2). There was no invasion or mechanical compression of aorta. There were bilateral bulky axillary lymph nodes measuring up to 5 cm. There were no findings of pulmonary embolism or compromised vascularity. The patient did not have any prominent neurologic deficit but was started on dexamethasone empirically due to concerns for possible cord compression.

**Figure 3:**
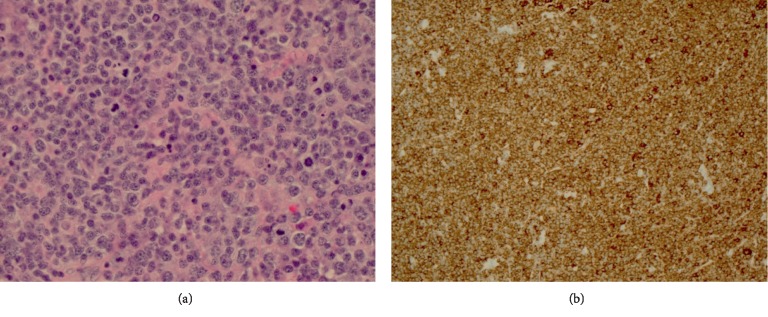
Immunohistological staining of the axillary lymph node: hematoxylin and eosin stain ×60 (a) and CD20 stain ×20 (b).

After a biopsy of an axillary lymph node, the patient was diagnosed with diffuse large B-cell lymphoma (Figure 3). Her FISH test was negative for double hit/triple hit lymphoma. She underwent bone marrow biopsy and lumbar puncture for CSF cytology. Results from both tests were negative for tumor infiltration into the bone marrow and CSF. Her PET/CT was negative for disease in the spleen and below the diaphragm. She was staged as stage II bulky lymphoma by Lugano modification of Ann Arbor staging system with NCCN-IPI score of 2.

The patient received 6 cycles of chemotherapy with cyclophosphamide, doxorubicin, vincristine, and prednisone plus rituximab (R-CHOP) and monthly intrathecal methotrexate infusion with each cycle of chemotherapy. Repeated PET/CT after completion of treatment showed no evidence of disease. She had one more repeat syncopal episode before treatment initiation, and her telemetry monitor more than 24-hour was uneventful. She had no further episodes since treatment. She denied any episode of dizziness, vertigo, or near syncopal episode during follow-up. The patient is currently awaiting consolidative therapy with radiation.

## 3. Discussion

Syncope is one of the most common presentations accounting for 1–3% of the total ED visits and admissions [7]. The differential diagnosis for syncope is broad, and making a diagnosis is often challenging. Syncope presentation as the first and the only symptom in malignancies is exceedingly rare. Only a few cases have been reported, and most of these cases were from patients with recurrent head and neck cancer [2, 3] or lung cancer [8].

Our case highlights an atypical presentation of DLBCL as recurrent syncope. The patient presented with episodes of syncope associated with fast recovery and no symptoms in between these episodes. Her syncope was initially confused with vasovagal syncope because of its similar characteristics and no other symptoms at baseline. The patient also did not have derangement in vital signs or electrolytes at presentation. Her further syncope workup including orthostatic vital signs, EKGs, later hospitalization under telemetry monitor, 2D echo, or CT brain and angiography was all normal.

To our knowledge, there are 7 reported cases of lymphoma presenting as syncope without direct cardiac involvement (3 cases of diffuse large B-cell lymphoma [4–6], 2 of primary mediastinal lymphoma [9, 10], and 2 of Hodgkin's lymphoma [11, 12]). All previously reported cases were found to have mechanical infiltrates or vascular compromise from the mass. Our case was interesting that the patient had an extensive mass in the anterior and the posterior mediastinum surrounding the descending aorta; however, both her CT angiography of head and neck and CT chest with contrast showed intact vasculature in the head and neck and also in the aorta. She did not have persistent bradycardia or hypotension, and there was also no apparent infiltration around the carotid body or around the aorta.

Many of the previously reported cases of malignancies presenting as syncope described similarities to vasovagal syncope as our case. The exact etiology of vasovagal-like syncope arising from underlying malignancies remains unknown. Literature review from previous similar case reports postulated that “irritation” of the vagus nerve might be the cause. A case series of syncope in lung cancer patients interestingly found that there was a higher incidence of syncope in patients with tumors located in the left hilum like our case [8]. This irritation also might explain that syncope is more prominent in patients with head and neck cancer with recurrence [13]. Our case had nonspecific lymphadenopathy along the left-sided carotid artery, which could be a potential explanation of the cause of syncope. Another possible explanation of etiology in our case could be syncope as an atypical, paraneoplastic, and systemic presentation as diffuse large B-cell lymphoma is known for, like B-cell lymphoma symptoms. A few cases have reported syncope as paraneoplastic neurologic syndromes in the past including Hodgkin's lymphoma, manifesting as autonomic failure [14, 15]. Then, this case might illustrate that syncope in malignancy, especially in lymphoma patients, may not have an obvious anatomical explanation, although there was no obvious electrolytes or hormonal derangement in the workup studies.

Our patient had no further episodes of syncope after her successful treatment with R-CHOP and 6 times of intrathecal methotrexate treatment, and she achieved full remission for her lymphoma. Case series of head and neck cancer patients with syncope showed that treatment of malignancy improved the syncope from 13 out of 17 cases [2]. Most of reported lymphoma cases also described successful treatment of syncope after treatment. Notably, our patient had a comparatively good short-term prognosis, despite extensive tumor burden from stage 2 bulky disease. In our case, syncope might be considered as a rare mass effect from DLBCL, which enables comparatively early presentation.

Here, we described a rare presentation of diffuse large B-cell lymphoma which was incidentally diagnosed while on workup for recurrent syncope. Syncope as the presentation of malignancy is rare and challenging to make as a clinical-anatomic correlation. We suggest that malignancy might be considered as a cause of recurrent syncope in the case of patients who have unexplained etiology, despite multiple investigations and of patients with significant history that raises concerns for cancers in thorax including lymphoma.
